# Virtual collaborative creative engagement in a pandemic world: creative connection for older adults with lived experience of dementia

**DOI:** 10.3389/frhs.2023.1223337

**Published:** 2023-12-15

**Authors:** Julia Henderson, Colleen Reid

**Affiliations:** ^1^ Department of Occupational Science and Occupational Therapy, University of British Columbia, Vancouver, BC, Canada; ^2^ Department of Therapeutic Recreation, Douglas College, Coquitlam, BC, Canada

**Keywords:** older adults, dementia, well-being, collaborative creative engagement, virtual, online, technology, community-based participatory research

## Abstract

**Introduction:**

Older adults were disproportionately affected by COVID-19, and isolation and loneliness became key risk factors for mental illness and decreased quality of life. Older adults with lived experience of dementia and their care partners experienced isolation, loneliness, anxiety and depression, already heightened due to social stigma. Reduced access to resources was a notable problem.

**Objective:**

This Canadian qualitative study investigates the *Raising the Curtain on the Lived Experience of Dementia* (RTC) Project's virtual turn in program delivery during the pandemic, asking “How did virtual collaborative creative engagement (CCE) impact well-being for people living with dementia and their care partners?”; and “What are key elements of RTC's unique virtual CCE approach?”

**Methods:**

The study employs reflexive thematic analysis to analyse interviews and focus groups with the project's artist facilitators, researchers, peer collaborators living with dementia, and their care partners. Findings: Themes describe key elements of RTC's unique approach to virtual CCE and include: “Adjusting Expectations and Adapting to Technology”; “Re-imagining Creative Engagement in Virtual Space”; “Sustaining Reciprocal Caring, Learning, and Support”; “Disrupting Stigma and Welcoming a Wider Audience”; and “Supporting Well-being through Empowerment, Community, and Creativity.”

**Discussion:**

Findings offer new perspectives on how virtual CCE not only has the potential to decrease loneliness and isolation and associated mental health risks for older adults living with dementia and their care partners, but also can work to disrupt stigmatizing representations of dementia, promote inclusion, and enhance citizenship.

## 1. Introduction

The COVID-19 pandemic provoked a mental health crisis for people of all ages worldwide. For older adults, who were disproportionately affected by COVID-19, and in some locations had self-distancing restrictions greater than other age groups, isolation and loneliness became key risk factors for mental illness. While some studies found older adults were at less risk of pandemic-related emotional distress than younger people (1), such findings are highly culture and context dependent. Many studies revealed that older adults were at increased risk of pandemic-related loneliness, which was associated with decreased mental health and quality of life (2, 3). Older adults also were shown to experience pandemic-related financial challenges associated with worse mental health and well-being (4). For people with lived experience of dementia and their care partners, the pandemic increased their risk of isolation and loneliness, and increased rates of anxiety and depression, which were already heightened due to social stigma. According to Chong et al. “Older adults living with dementia in the COVID-19 world have experienced reduced access to support and activities. These changes have caused distress and exacerbated behavioural and psychological symptoms of dementia” (5).

In the early days of the pandemic, many organizations providing services to people living with dementia believed that it would not be possible to offer services online, thinking that a diagnosis of dementia would disrupt people's ability to meaningfully participate, and also adopting the ageist belief that older people (including spousal care partners) would be incapable of handling technology. However, the community-based, art-engaged 5-year research project, *Raising the Curtain on the Lived Experience of Dementia* (RTC) located on the Sunshine Coast in British Columbia, Canada, pivoted almost immediately to continue its creative engagements with people living with dementia in a virtual context. This study uses reflexive thematic analysis (6, 7) to analyse interviews and focus groups with the project's researchers, artist facilitators, individuals with the lived experience of dementia (called peer collaborators within RTC), and their care partners. Themes describe key elements of RTC's unique approach to virtual collaborative creative engagement (CCE) with people with lived experience of dementia and their care partners which was developed in response to the pandemic.

### 1.1. Literature review of related works

The COVID-19 pandemic had disproportionately negative consequences for older adults broadly. In addition to “excess mortality,” (8) these included,

the presence of psychological symptoms, exacerbation of ageism, and physical deterioration … decreased social life and fewer in-person social interactions … occasionally associated with reduced quality of life and increased depression. Difficulties accessing services, sleep disturbances, and a reduction of physical activity (9)

For people with lived experience of dementia and their care partners, these negative consequences were often exaggerated. Dementia is an umbrella term that refers to a set of symptoms including memory loss, changes in mood, and difficulties with thinking, problem solving, and language (10). In Canada, the location of the study, over 597,300 people are currently living with a dementia diagnosis. This number, prior to the pandemic, was expected to increase to nearly 1 million by 2030, and more than 1.7 million by 2050 (11). While dementia is typically associated with various neurodegenerative conditions (12), rather than named as a mental health diagnosis, its impacts on mental health and well-being have been well documented. People living with dementia commonly experience stigma and discrimination (13–15), disempowerment (15), depression (16), and “struggles with self-identity, independence, control and status, participation in meaningful activities, and how to view the future” (15).

The COVID-19 pandemic presented global health, social, economic, and environmental challenges that put both people with lived experience of dementia and their care partners (also called caregivers or carers in various studies) at increased risk for physical and mental health challenges. Based on large-scale international clinical data, Numbers and Brodaty suggest that “As well as being at increased risk of contracting COVID-19, older adults with dementia are also more likely to have more severe disease consequences than those without dementia” (17) A range of studies considering data from Europe and Australia found that the experiences of social restrictions and isolation have been associated with worsening cognitive decline for people living with dementia (18), worsened neuropsychiatric symptoms such as depression (19), apathy (19, 20), delusions (19), anxiety (19–21), irritability (19, 20), agitation (19), and loss of self-worth and purpose stemming from inability to participate in meaningful activities” (21). In British Columbia, Canada, Tam, Dosso and Robillard found that both people with dementia and their care partners reported increased levels of stress and feelings of isolation resulted from COVID-19 (22). Amongst care partners increased stress and reduced social networks were associated with uncertainty about the future and loneliness in Australia, Germany, Spain and the Netherlands (19), and, in the Italian context, increased levels of depression were associated with increased caregiver burden during lockdown (23). In a large sample of family care partners in the UK, 43.7%, of them reported moderate loneliness and 17.7% reported severe (24). Survey data from a Canadian study by Faieta et al. showed that 87% of dementia care partners who were separated from their loved one during COVID-19 social isolation experienced negative mental health consequences beyond typical contexts, and over 70% of care partners were concerned with the care their loved one received (25).

The stigma of dementia has long been considered responsible for many of the negative experiences of people living with dementia and their care partners (26). Dementia stereotypes, that construct dementia as a tragedy (27, 28), pathology, moral failing (28), death of self, and social terror (29), have been widely associated with loss of identity, personhood, and citizenship (27, 30–32). In response, in recent years, human rights-based approaches that create opportunities for citizenship are favoured in Dementia Studies (27, 32, 33). Participatory arts-based practices have grown in popularity because many readily complement and integrate a citizenship orientation. Engagement in participatory arts is reported to positively impact health, well-being, social connectedness, and quality of life of older adults, including older adults living with dementia (34–38) While many participatory arts programs have focused on the beneficial role of the arts in enhancing health, wellbeing, or quality of life, increasing attention is being given to their role in supporting citizenship for people with lived experience of dementia. Canadian researchers Dupuis et al. demonstrated that, for people living with dementia, engagement in the embodied practice of participatory arts alongside family members, visual and performance artists, and researchers, profoundly challenged the tragedy discourse of dementia and fostered narrative and relational citizenship (27). Phinney et al. illustrated how participation in a community gathering and co-created performance at the American/Canadian Peace Arch (which included social gathering, a shared meal, exhibition of artworks by people with dementia, live music and participatory dance, tai chi instruction, and the creation of a community quilt) extended social citizenship by “challenging discourses and practices that maintain boundaries between ‘us and them’” (39). Through critically examining elder clown practices for artistically engaging with people living with dementia in care home settings, Kontos, Miller and Kontos proposed a model of relational citizenship that “extends the concept of social citizenship by presuming that support of the central tenets of relationship-centred care …and embodied selfhood theory are necessary to more inclusively grant citizenship entitlements to persons living with dementia in long-term residential care” (32). To the authors' knowledge no studies to date link *virtual* participatory arts to concepts of citizenship.

Beyond considerations of citizenship, new research on participatory arts has begun to focus more specifically on the unique benefits of collaborative programs that “engage the innate creativity of people with a dementia” (40). Dementia scholar and playwright Anne Basting has been seminal in developing and implementing participatory arts approaches that integrate inclusive and collaborative ways of working together and highlight the creativity of people living with dementia (34, 35, 41–43). Basting's many community-engaged projects employ what she names “creative care” (34)—an approach grounded in the belief that creativity is universal, exists in everyday moments, and emerges through relationships. The concept of co-creativity, used by the UK project *Created Out of Mind* (44) is similar to Basting's creative care approach. Zeilig, West & van der Byl Williams describe co-creativity as a nascent term that refers to collaborative approaches to creative engagement that are: innately democratic and non-hierarchical, interactive and relational, dialogic and empathetic, recognize diverse capacities of all those involved and weave them into the creative process, and rely on and create openness, receptivity, and imaginative space (40). Kontos, Grigorovich & Colobong address the theoretical underpinnings of collaborative, creative practices for working with people living with dementia, arguing that “creativity is not an individual cognitive trait but rather emerges from the complex intersection of enabling environments and the embodied intentionality of all involved” (45). They critique the notion of creativity as a form of genius that is uniquely the domain of artists, and [similar to Basting (34) and Zeilig, West & van der Byl Williams (40)] advocate for a broader view of creativity that “account[s] for the everyday and ordinary creativity of ‘regular’ citizens, including persons living with dementia” (45). Kontos, Grigorovich & Colobong further call for scholars who are adopting embodied and relational approaches to creativity to more fully engage with theoretical and empirical scholarship, “either in terms of understanding how the body is a site for the inscription of discourse and the making of particular subjectivities, or in terms of how capacities, senses and experiences of bodies are central to the exercise of human agency and citizenship” (45).

The COVID-19 pandemic presented a profound challenge to arts-based, co-creative projects that worked with people with dementia and relied on collaboration, relational support, experimentation, extended time together, and embodied practices. Many such projects had to make a quick decision about whether to transition to virtual delivery, with little research to support its effectiveness, efficacy, or recommend best practices. As well, limited infrastructure, resources, and guidelines to support implementation were available. Since the “pandemic pivot”, research has begun to emerge about online programs for older adults, including people living with dementia. Despite widespread prejudice around older adults that predicted their limited capacity with technology, older adults are reported to have used digital technology during COVID-19 to connect socially, access resources, and manage isolation (46, 47). A systematic review by Rai et al. of digital technologies specifically for people living with dementia, reported evidence that “technologies hold potential to improve quality of life and reduce isolation/loneliness for people with dementia” (48). Further, MacRitchie et al.'s scoping review of research on technology-assisted creative arts activities for older adults living with dementia, found technologies were mostly designed for music activities (listening, and music-making), storytelling and visual arts. Devices used included applications for tablet or computer, portable media players, video game systems, Virtual Reality (VR) and online software, thus demonstrating people with dementia were capable of engaging with a broad range of technologies. The majority of devices were reported to be custom-made and at the prototype phase (and not commercially available). Their recommendations for future research were to involve people living with dementia increasingly in co-design, to progress device development past prototyping, and to investigate comparisons across devices and across arts-based activities (49). Canadian researchers Faieta et al. (25) found that amongst care partners separated from their loved one with dementia during COVID-19, the majority perceived the need for smart mobile device (SMD) app use—a particular kind of digital technology—for example, video conferencing apps, messaging apps, browsers, etc. They found “more limited SMD app use was associated with poorer mental health outcomes” for care partners (p. 6). Further, a Texan qualitative study (51) of virtual memory cafés for individuals with Alzheimer's and related dementias and family care partners during COVID-19, found the virtual cafés offered important benefits including: “reprieve” (p. 4) from daily life and the stressors of the pandemic, a reminder of “what is still possible” (p. 5) despite living with dementia, a sense of “connectedness,” “belonging,” (p. 5) “community” (p. 6), and “inclusivity,” as well as “cognitive stimulation,” “education,” and “resources.” (p. 7) The researchers argue that virtual models may support social connectedness beyond the COVID-19 context, but that attention must be paid to the issues of access to technology and limitations of virtual engagement for those with late stage dementia (50). These studies point to the potential benefits of digital technology and virtual participation for people with lived experience of dementia and their care partners, and suggest the need for further research around access and implementation.

In terms of virtual *arts-based* programs, art workers in the UK reported that remote delivery of arts activities helped people with dementia gain a sense of community, find structure and purpose, combat physical isolation, and contribute and share, though they noted that setup and maintenance could be issues to access, and that some people with dementia could experience challenges interacting through technology (51). A Brazilian intergenerational virtual participatory arts program, Playful Living, designed to promote wellbeing and social connection among vulnerable older adults (mostly with aphasia and dementia), reported 83.7% adherence suggesting feasibility and acceptability, and noted that participants reported feelings of social connection and a sense of having learned together (52). Thompson et al. used interpretive phenomenological analysis and collaborative song writing to analyze participants' experiences with a virtual format in an Australian therapeutic community choir for people living with dementia. The experience reportedly “was acceptable, provided relief from the stress of COVID-19, and kept members connected” (53). However, technological limitations, such as accessing and learning new technology, and having the right equipment and someone to help them learn to use it were noted as challenges (p. 19). Research on virtual arts-based programs for people living with dementia and care partners remains quite limited, however, and most studies describe the experiences of participants, but not the “how to” aspects of the engagement. One exception is Kontos et al.'s qualitative study of participants with dementia who took part the Canadian Sharing Dance Seniors program, which involved remotely streamed dance sessions in long term care and community settings (54). The researchers provide details of how the program unfolded involving on-site facilitators and an emphasis on creative expression. They also identified and gave specific examples of how, in response to virtual instruction, persons living with dementia showed intrinsic capacities for creative self-expression, including playfulness and imaginative verbal and nonverbal engagement. According to the researchers, the participants “co-constructed and collaboratively animated the narrative of the dances,” participating with playfulness and sociability (p. 714). However, while instruction was virtual, participants took part in face-to-face groups, so they were not collaborating with each other virtually. While much research on programs developed throughout COVID-19, no doubt, forthcoming, a recent Baring Foundation report, *Creative Aging in Lockdown and After* (which provides numerous case studies of online arts initiatives for older adults), highlights the need within the creative ageing sector “to innovate and adapt through researching, refining, documenting and disseminating new ways of working,” with older adults through technology (55). This article represents a novel contribution to this important, emerging area of research.

## 2. Methods

### 2.1. Study context

*Raising the Curtain on the Lived Experience of Dementia* (RTC) was a 5-year (2017–2022) research collaboration between three partners: Douglas College in Coquitlam, B.C., Canada (education), Good Samaritan Society's Christensen Village, a long-term care home in Gibsons, B.C. (health care), and Deer Crossing the Art Farm in Gibsons, B.C. (participatory arts). The RTC team was comprised of researchers (academic researchers, a postdoctoral researcher, and research assistants), artist facilitators (or AFs, who were professional artists with experience in community engaged arts), individuals with the lived experience of dementia (called peer collaborators within RTC), and peer collaborators' care partners (who were their spouses or children). In RTC we sought to answer two research questions: “What is the lived experience of dementia?” and “How can participation in collaborative creative engagement (CCE) enhance the well-being of people living with dementia, their family and caregivers, and our society as a whole?”

RTC was guided by a community-based participatory research framework (CBPR). CBPR operates from a value base of sharing power and resources, and working for beneficial outcomes for all participants (56). It aims to foster equitable community engagement and active citizenship (57) through utilizing unique partnerships, methodological innovation, and community engagement in the co-creation and co-mobilization of knowledge to address issues of importance to community members (58–60). In CBPR, diverse perspectives (e.g., artistic and scientific) stimulate and inspire each other and enrich processes of collaboration (61).

All of RTC's project activities fostered the iterative interplay between CBPR and collaborative creative engagement (CCE). CCE is a “social and embodied experience of meaning making.” (62) because it unites cognitive processes, creative probing and expression, and emotional experience (62). It is similar to the creative care (34) and co-creativity (40, 44) approaches described previously in the literature review. The interplay between CPBR and CCE was driven by and supported our commitment to create inclusive communities for individuals living with dementia, to build collaboration between all team members, to value different kinds of knowledge, and to create spaces for advocacy and social action.

RTC began by collecting data about peer collaborators' lived experience of dementia through a focus group. Themes derived from the focus group data were then used to structure the first “round” of CCE workshops. Data gathered from the first round of CCE workshops were used to further refine and develop the themes. The CCE workshops were collaboratively planned with input from peer collaborators. They were run by AFs using diverse media (for example, theatre, music, visual art, new media), but leadership was shared across the team, and researchers also attended. Over the 5 years, RTC's implementation of CCE varied between large and small groups and between in-person and online delivery. Between September 2017 and March 2020, RTC held 40 2-h in-person CCE workshops with peer collaborators that did not include care partners, and two face-to-face performances for invited audiences. For a more detailed description of these CCE workshops, see Reid, Landry & Henderson (63). In March 2020, pandemic public health orders suspended in-person CCE workshops. The team collaboratively made the decision to continue CCE remotely; from April 2020 to February 2022 all project activities were distanced, involving Zoom, and (for two peer collaborators and their care partners) several telephone sessions. Moving RTC online involved a significant amount of planning and an amendment to our ethics protocols. Moving the project to Zoom (primarily) enabled the continuation of CCE workshops, however it also involved greater exposure to the homes, family and friends of team members and research participants. We remained mindful of the blurring of these boundaries and careful in our documentation of the CCE sessions.

To mitigate concerns about peer collaborators and care partners navigating technology, becoming confused or fatigued online, and lacking necessary art supplies, the RTC team changed the structure of CCE. Now each AF was paired with one peer collaborator and their care partner, and a student research assistant (RA) supported as needed. AFs, peer collaborators, and care partners met in smaller groups, usually with two peer collaborators, their care partners, and their AFs. Workshops became more focused on individual interests, rather than the whole group doing the same activity as had been the case for in-person CCE. This phase of the project included over 120 1-h virtual CCE workshops, and two virtual performances where researchers also collaborated with AFs and peer collaborators (64, 65). The virtual CCE workshops were recorded on Zoom alongside the documentation of fieldnotes by the student RA in attendance. After each workshop the student RA reviewed the recording and added portions of the verbatim transcript into the fieldnotes that were relevant to the broader research question and that upheld our team's ethical commitments (Team members agreed that full verbatim transcripts were unnecessary but that excerpts that were relevant to the primary research questions added depth and accuracy). From March to June 2022 RTC re-introduced in-person engagements that adhered to the public health mandates, in conjunction with continued online sessions. In these last months of the project RTC held five in-person CCE workshops and hosted two days of live-streamed hybrid performances and presentations in which all team members participated (66, 67).

### 2.2. Research design and methodology

While the overall goals of RTC were broader, this article represents a more focused sub-study of the project's transition to virtual CCE. It was inspired as we coded data collected throughout project's virtual transition that suggested the relevance of this topic. This sub-study explored the research questions: What are key elements of RTC's unique virtual CCE approach?; and, how did virtual CCE impact the well-being of our peer collaborators living with dementia and their care partners? The sub-study used a qualitative design, informed by the principles of CBPR. In CBPR clear distinctions between researcher and research participant become untenable in the context of peer research where “researchers are known to participants and do not always leave ‘the research field’ when the project is over” (56). Because RTC was a lengthy, collaborative project in a small community, team members were embedded in and contributed to the data gathered. However, we remained consistently committed to our own training and reflexivity, both of which were deeply integrated in our ethical commitments, research processes, and bi-monthly team meetings. In this sub-study we used reflexive thematic analysis (6, 7) to develop trustworthy, rich descriptions that reflected participants' verbatim descriptions of the processes of virtual CCE and its influence on peer collaborators' and care partners' sense of well-being.

#### 2.2.1. Data collection

This sub-study used qualitative data that had been collected in RTC for broader purposes, and analysed it to answer the specific research questions of the sub-study. The data set involved 5 semi-structured interviews (30–90-min in length) with 5 researchers, 23 interviews with 8 AFs, and one focus group with 5 AFs. While the data set for the broader project was larger and more equitably represented all voices within the project, this data set gave greater voice to AFs because of their central role in organizing CCE sessions, and because their experience as professional artists meant they could speak to the artistic processes and techniques beyond a lay understanding. The AFs participated in three sets of interviews: the first shortly after the pivot to online engagement (summer 2020); the next slightly later during virtual CCE activities (winter 2020 to spring 2021); the final near the end of the project (spring 2022). In order to represent the voices of peer collaborators and care partners, especially as related to their experiences of well-being, we used 11 interviews that were conducted with them by AFs during four livestream online performances for public audiences. We also used one care partner panel discussion, also from a livestream performance. These data were chosen over conducting additional interviews with peer collaborators and care partners because out-of-context interviews were difficult given peer collaborators' experiences of short-term memory loss, because the team had jointly chosen to concentrate energies on public sharing activities, and finally, because in the pandemic context, additional research interviews felt burdensome to peer collaborators and care partners. More details of the data set appear in Table 1.

**Table 1:** Data set.

Data	Number	Conducted by	Date	Context
Interviews with researchers (*n* = 5)	5	RA	Winter 2020	Online
Interviews with AFs (*n* = 8)	23	RA (8)	Summer 2020	Online
		First author (7)	Winter 2020 to spring 2021	
		RTC researcher (8)	Spring 2022	
Focus group with AFs (*n* = 5)	1	First author	Summer 2022	In person
Panel discussion with care partners (*n* = 6)	1	2 RTC researchers and 1 AF	Spring 2022	Hybrid conducted during livestream public performances (58)
Interviews with peer collaborators & care partners (*n* = 9 PC & 9CP)	11	AFs	Spring 2020 (55, 56) and Spring 2022 (57, 58)	Conducted online (55, 56) and hybrid (57, 58) during livestream public performances

AF and Researcher interviews and the AF focus group, used semi-structured interview guides, including open-ended questions such as the following: For you, how is the experience of working online/virtually? What changed because of COVID-19? How did changing the format from in-person CCE to virtual engagement change your creative process or practice? What was lost and what was gained? What is needed to maintain continuous and meaningful engagement? The online peer collaborator panel was also guided by semi-structured questions about experiences with RTC and virtual CCE. Interviews with peer collaborators were more open-ended around a collaboratively pre-chosen topic (for example, discussing a particular artistic creation), and responsive to peer collaborators' in-the-moment interactions.

#### 2.2.2. Demographics

Of the 31 participants in this sub-study, 24 team members lived in the local community, 6 lived in a larger city 45 min away, and one lived in another small distanced community. Twenty-three of the team members had been involved with RTC since its inception in 2017. Across 8 AFs, artistic expertise included: theatre, visual art, film making, podcasting, photography, web-design and new media. The 9 peer collaborators ranged in age from their 60's to 80's. They had diagnoses including Alzheimer's Disease, Vascular Dementia, and Lewy Body Dementia. While all peer collaborators had early-stage dementia when they began RTC, at the time of this sub-study the stage of their disease ranged in severity. Eight named English as their first language; one named German. Three care partners were children of peer collaborators, 6 were spouses (of these, two were same sex spouses). Eight peer collaborators lived in the community with their care partner(s), and one lived in long-term care where their care partner also lived. Two couples chose to do some of their CCE sessions by telephone. The others did sessions by Zoom and occasionally FaceTime. The devices used by peer collaborators and care partners were as follows: 5 pairs exclusively used iPads (of these one pair had the iPad connected to a larger monitor provided by RTC); one pair exclusively used a desktop computer; one pair used a combination of iPad and phone calls; one pair began with a combination of desktop computer and phone calls but due to technical difficulties transitioned to an iPad provided by RTC; one pair used an iPad for large group sessions and performances but chose not to participate in small group CCE sessions.

#### 2.2.3. Data analysis

To remain consistent with our CBPR framework we used a collaborative approach to reflexive thematic data analysis (6, 7). All recordings were transcribed verbatim by research assistants or the first author. Pseudonyms were used to de-identify data in all Researcher and AF interviews and the AF focus group. Because the peer collaborator and care partner data came from transcripts of public livestream recordings, in which they chose to use their real names and consented to have permanently posted on YouTube, their real names have also been used in this article. Verbatim transcripts were analyzed using the software programs NVivo 11 and MS Word. The first author (a postdoctoral researcher on the project) was not able to access a computer with the NVivo 11 license due to social distancing, but having previously used NVivo, was able to replicate a similar style of coding using MS Word. Therefore, the coding style and processes for all researchers were aligned. Researchers immersed themselves in the data, reading and re-reading the transcripts, reviewing reflexive fieldnotes, and relistening to recordings when necessary. Initial “codes were descriptive and closely reflected the data. The researchers used constant comparison to compare and contrast codes, grouping and regrouping them into higher level codes. Initial coding of about half of the interviews was done by RAs with oversight by the article authors. Most of these RAs had participated in the CCE workshops and recorded the fieldnotes. After initial codes were collaboratively decided, a code book was created to guide the RAs and create consistency. The authors and an RTC researcher met regularly with the RAs to discuss and compare coding, and to adjust the code book to incorporate their insights. The other half of the interviews and the focus group transcripts were coded by the first author, who then compared and contrasted this data with the data coded by RAs to create conceptually higher-level codes and overarching themes. This allowed for the analysis to reflect the relationships between the codes and broader themes and achieve rich descriptions of participants” experiences (58). To establish trustworthiness, the first author engaged in member checks by email and the focus group with the AFs; this involved presenting coded data, asking for feedback, and making adjustments to make sure AFs felt it reflected their insights. Throughout the project coded data was also shared with peer collaborators (and later care partners) through creative engagements and refined based on their responses. Triangulation across participant groups was also used to enhance the credibility of the data, and the multiple perspectives of the RAs and the two authors also helped confirm the codes and themes reflected the data. The first author used a reflexive journal to record and reflect on assumptions and consider how they might influence the analysis process, and she also kept and reflexively analyzed detailed analytical memos. The second author followed the audit trail and reviewed coding to make sure decisions reflected the perspectives expressed by participants.

## 3. Results

Five themes were identified. The first four describe the processes of CCE. The fifth details the peer collaborators' and care partners’ experiences of well-being related to CCE engagement. In these theme descriptions, the following acronyms are used to indicate roles of speakers: AF = artist facilitator; R = researcher; PC = peer collaborator; CP = care partner.

### 3.1. Adjusting expectations and adapting to technology

Quite early in the COVID-19 pandemic, when social distancing orders came into effect in British Columbia, RTC made the choice to shift its activities online. As Adam (AF) described it, “going virtual” meant that “we as a team and myself personally, had to adjust expectations and adjust the kind of engagement or exercises we would do.” In some cases, shifting to virtual team meetings facilitated participation for team members at different locations. Charlotte Rae (R) noted,

Because of COVID so much of the project was transitioned to being virtual that I was able to participate to a much greater degree so for me it was actually a big benefit as it meant that I could take part in the project in a more central way.”

#### 3.1.1. Including care partners

Working together from a distance brought challenges and revised expectations. One of the biggest changes to the project was the involvement of care partners. When RTC met in person prior to the pandemic the CCE workshops provided a space for peer collaborators to have independence from their care partners, and for care partners to have a break in their caring responsibilities. Now involving care partners was necessary to help peer collaborators manage the technology and scheduling. As Sonia (AF) noted, “after we went online, we became a bit reliant on care partners to act as co-facilitators, and especially in the tech and getting the person [peer collaborator] to be present, physically present.” Esme (AF) elaborated on the new learning required for care partners: “A big part of it was dealing with the technology itself … I mean her daughter was the person who would set everything up for us but it was new for her as well.” Despite the challenges and loss of time apart for peer collaborators and care partners, involving care partners in creative engagements had strong benefits, as DD (AF) described:

there's probably lots of moments where the zooming really, really worked. It created a forum for us to bring the partners in … the caregivers. And I think that's a nice progression of this project. And I think it's supported the partners in ways and made them feel more a part of that, rather than, you know, they drop their partner off. They're engaged … and challenged.

Involving care partners and connecting via technology required adaptability, as it meant new demands on everyone.

#### 3.1.2. Developing caring and supportive virtual practices

The team also was compelled to develop caring and supportive approaches to virtual practice. As James (AF) put it “the team making that switch to … going online, it developed a whole new set of skills.” The team had to spend more time and develop ways of providing technical support. Elizabeth (AF) described this, “I feel like we did a lot of back and forth to try and help get our participants online, like we cared a LOT.” One of the researchers spent more time “touching base with people, reminding them, offering emotional support and encouragement regarding the technology” (Charlotte Rae, R). Jade (R) described the type of support that was needed, “support for the simplest things with technology, moving to a new device, how do you change your volume?” Elizabeth (AF) recounted driving to a peer collaborator's house (once social distancing orders allowed outdoor visitation) to help troubleshoot from outside,

I went to his house…to try and troubleshoot his Wi-Fi and where it would reach and I think he had the wrong passcode… I got my other sheet of passwords… I think we did give him an iPad to support that and then tech support.

In some cases, the team provided peer collaborators with devices to use. Strong support was required, as Elizabeth (AF) put it, “for a lot of people [peer collaborators and care partners], the technology was a really scary thing to go into, it was really stressful for them.” Thus, the team put much thought and effort into making the experience as approachable as possible.

For AFs, switching to online with little notice meant they were learning how to use virtual tools effectively at the same time as helping peer collaborators and care partners adapt to Zoom. As Esme (AF) expressed, this was a steep learning curve, “I had never done a Zoom conference much less FaceTime or anything like that. I don’t have a huge facility or aptitude for technological things. So that's been a bit of an uphill challenge.” Even for AFs who had more facility with technology, changes in how they prepared for CCE sessions were required. Ki (AF) described how virtual CCE required their workshop planning and preparation to be more methodical and purposeful:

Having one of the participants that speaks the least, I would spend a large amount of time in between sessions trying new things, try[ing] to be more prepared. Look over my video from before, … how can I make this so that she feels comfortable? And she's not so alienated by this technology … that she doesn't get any benefit out of it.

Participating in long Zoom sessions was difficult for peer collaborators, Louise (AF) described, “This Zoom wears them out after like an hour tops, they're done.” But Zoom fatigue was not unique to peer collaborators. Participating in Zoom sessions could be challenging for all team members,

A Zoom call for anyone for two hours, I think can be hard on the brain and the psyche and everything. It's quite draining. So, I think making adjustments and keeping our timeframes short has been more successful in transitioning to an online space (Elizabeth, AF).

Thus, the team shortened the CCE sessions to 1-h, and used smaller groups. Sonia (R) noted “when we broke up into smaller groups … it really highlighted just how meaningful the work was to the individual [peer collaborators], because they were so willing to work with us” Adam (AF) further elaborated that Zooming with smaller groups was “probably the most exhausting, but also the most rewarding part, cause you were developing this really strong relationship.” To accommodate fatigue, the team also adjusted the pace of CCE sessions. Adam (AF) explained: “It's certainly slowed it down even more because it had to be … more methodical and creating simple exercises that could translate to a virtual engagement.”

With this came certain losses, particularly in casual interactions. Ki (AF) lamented that “it just wasn't the same as being in a whole room with chatty, interesting people who are excited to be with one another.” But as Sonia (R) pointed out, virtual engagement also brought “a whole new level of intimacy with our participants, to be in their homes, to see their interactions with their partners, to be spending so much one-on-one time with them.” DD (AF) also detailed how working in smaller groups meant that peer collaborators received more individual attention, “we are one-on-one… it became much more specific and catered to their strengths, or the world that we know they like to resonate in.” AFs noted some benefits in terms of their own accessibility; “I can actually be more focused for an hour” (DD, AF). Virtual engagement also made participation more accessible for people who lacked transportation,

The one thing we gained was that we didn't have to deal with the headache of transportation… it was just a challenge in a rural community like ours where there is not a ton of supportive transportation… so we gained in terms of reach and connection to people. (Adam, AF)

It also made it possible for family collaborations to develop. In the case of Traudi (PC), she and her husband (a poet) and daughter (an aerialist and dancer) created a piece together with the assistance of their AF, which became part of the project's Backstage Pass virtual performance shared with a public audience. Her daughter described the experience as “very emotionally nourish[ing] … very connecting and maybe a little cathartic.” But not everyone was able to adapt. DD (AF) described how some peer collaborators and care partners stopped participating in creative engagements, “I believe two of our participants haven't participated in this last round, specifically because of the challenges with Zoom. Whether it's technological or the feeling of connection, they aren't playing with us right now.”

### 3.2. Re-imagining creative engagement in virtual space

The shift to virtual CCE not only created the need to support technology use, but also called for the development of new approaches to facilitating, inspiring, and supporting collaborative creative work. This meant drawing on, adapting, and expanding some of the project's previous approaches. Adam (AF) described how this process was both challenging and rewarding,

As we were trying to design activities collectively, … exercises and activities that would work online… I think it really stretched and tested my own ability to be adaptable… in some ways, I think that that was the gift of the Zoom era.

#### 3.2.1. Fostering feeling in virtual space

The first adapted approach the team developed to support virtual CCE was “fostering feeling in virtual space.” The project previously had a strong orientation toward drawing upon feeling as a way for participants to connect with each other, and as a gateway into creative exploration. Feeling each other's presence was key to group cohesion and creative exploration. Esme (AF) noted how she could feel “the joy … coming off the group when the group can come together.” Focusing on affect when working with people living with dementia can be effective, described James (AF), because “people don’t remember what you said, they remember how you made them feel.” Sandra (CP) lauded this approach within RTC,

the feeling of belonging and being amongst friends and the stimulation from the creative engagement lasts long after … having been in the moment and having been thinking and using your imagination and having fun,

Fostering feeling in virtual space, though, did not come as readily as it did in person. Adam (AF) explained,

I come from a live performance or live art background so a lot of your time, energy, thought goes into creating a room, creating a feeling in a room, something in a physical space, so … how do you create that when people are in different spaces?

One technique the team continued to favour in online interactions was “being present” or “being in the moment.” James (AF) explained that “it's not trying to draw on memory all the time, [it's] looking at the moment, and working with the person in the moment.” Louise (AF) emphasized that being in the moment “remove[d] any shame,” because “it wasn't about that [remembering or feeling a certain way], it's just being present.” This adaptability enabled the peer collaborators to exercise more control or citizenship in those moments. Sandra (CP) described the value this has for her mother,

sometimes my mom is concerned that she's going to do something wrong or she's not going to get it right. That doesn't happen here because …no matter what happens, a spontaneous break into song as she does routinely, everybody just joins in, it's applauded and you know people encourage that.

Encouraging this type of presence and adaptability was not only something AFs tried to inspire in peer collaborators and care partners to help them feel at ease, it was also an approach AFs and Researchers adopted themselves.

It's really being in the moment with our participants and recognizing when something is making our participants uncomfortable and being willing to let go of the creative activity that we were hoping to achieve… really allowing the participants to turn the direction of the creative activity with their ideas and with their feelings at any time (Elizabeth, AF).

Attending to breathing was helpful to achieving this type of presence. Louise (AF) explained, “Allowing yourself to go into it with full relaxation and breath is so essential in allowing ourselves to play.” As a result, RTC incorporated breath exercises into some of its online activities within team meetings and creative engagements. They were integrated into the project's collaborative song writing practice too.

One online-specific technique the team found supported being in the moment was avoiding the blurred backgrounds commonly used on Zoom. This allowed participants to see and respond to each other's actual social and physical contexts.

I think on Zoom meetings … the past history of it was to pretend that your background reality doesn't exist, like some people will blur it out and try to have it with the plain background and it's just like, no, I'm here. I'm present… like kind of breaking that Zoom conference energy by integrating the space that everybody was occupying (Elizabeth, AF).

Avoiding blurred backgrounds allowed for the sharing of more intimate, personal space. As a result, the collaborative teams drew inspiration from home spaces to engender positive feelings. Ki (AF) explained that seeing her peer collaborator's home over Zoom was an “opportunity” to be influenced, in the moment, by the objects in her environment,

I could also tell on the Zoom calls that there were like many examples of her artwork because she was a ceramicist, potter, and she made all manners of types of pottery, including visual art … that's framed and hanging on the walls … it's just really extraordinary the breadth of her artistic skill.

This inspired a shared activity, Ki (AF) explained, “their next photo assignment was to take photos of these [lamps she made], and then show me. And so we both did our thing and we came together. And then we made a photo collage of their lamps and my lamps.”

Acknowledging and validating feelings was also important to encouraging emotional expression. Humour was used often as a means of connecting. Adam (AF) recalled the great sense of humour of one peer collaborator, “I remember he… would laugh every time… We just set him laughing. And it was like a bubble burst in the room. And then … it was just so freeing.” However, fostering feeling was not only about encouraging fun, humour, and joy. It also involved acknowledging and validating peer collaborators and care partners' more difficult feelings, and accepting them “where they were at” (Ki, AF). DD (AF) expounded, “if they are grumpy, they are grumpy and if they’re angry about their situation, they are angry.” He noted that one of his mistakes in the beginning was to try to make everyone happy, saying “you don’t have to do that and it's okay… eventually we just find our way out of it or we explore it.” In virtual space, in the pandemic context, fostering feeling sometimes meant acknowledging that Zoom brought frustration, and that, as Adam (AF) put it, “Zoom fatigue, online time, added to our… stress in the sense of disconnection.” Sometimes it meant acknowledging the feelings of isolation and loss people felt about not being able to meet in person. Esme (AF) described one peer collaborator, “She would also say she just missed hanging out with everybody,” and “every week she would ask when are we going to be able to get together again in person.” Sometimes fostering feeling meant accepting the confusion peer collaborators experienced about the pandemic and trying to offer comfort. DD (AF) described how one participant thought everyone was meeting without her,

I would witness how the participant I was dealing with couldn't remember what's going on. She was often wondering how everybody is and assuming that everybody else is getting together in the room that we always get together but she wasn't. So, it was hard to witness her lack of comprehension and her desire for connection.

“Fostering feeling in virtual space” helped the team stay connected and helped sustain meaningful engagement despite the difficulties involved in collaborating online. Esme (AF) recognized how “feeling” allowed for authentic connections, “I think a true strength of our project was the bonds that people made with each other … in that they can witness each other going through a shared experience.” Fostering feeling was also a technique used to connect with virtual audiences during the project's public live stream events.

#### 3.2.2. Inspiring virtual play with intentionality

Building on techniques for fostering feeling, especially being in the moment, the next approach that was key to the project's effective CCE, was “developing virtual play.” Similar to fostering feeling, play had always been a key element to RTC's creative engagement sessions. DD (AF) described the accessibility and effectiveness of engaging from a place of play,

I didn't know anybody who ultimately wouldn't get into the play. Because again, it's play. And if we take out the stigma of “kids do that” … and just make it more about the fun. And there's a lot of things for them to choose, then they start getting into it.

Even peer collaborators and care partners who did not consider themselves “artistic” were able to meaningfully engage in play. Louise (AF) described the success of the team's play approach with one hesitant peer collaborator,

She still is hesitant to be doing anything artistic… [saying] “I'm not an artist,” because she was told she wasn't at a very young age… she came up with … just beautiful, beautiful, beautiful work of expression. And so there's so much in that … allowing them to explore allowing.

Play was often invigorating by way of its novelty, Elsie (R) noted:

I think the … importance of play in RTC is that it makes you more alive when you play… it's like play offers a new experience that is very opposite to what often people might feel or might experience in their day-to-day lives, when they are living with dementia.

Sandra (CP) felt that RTC's playful orientation was central to the positive experiences her mother had with the project,

It's not just the talking … I think that the collaboration and the fun and the encouragement to use your imagination and your intelligence … and the love, I think there needs to be more opportunity for that kind of thing.

However, like “fostering feeling,” play was more challenging to evoke online. Esme (AF) noted that, “if we were meeting in a room, there’d probably be a bit more play.” The team consciously adapted and developed playful approaches for a virtual context. Some of the session warmup activities became more “conversation, check ins” (Esme, AF). The team also found playful ways to adjust warmups, which began each CCE session, to work on Zoom. A favourite warmup was Energy Ball which involved imagining a ball of energy and miming holding it, describing and miming how it changed, and passing it on to another person. Esme (AF) recounted the playfulness and acceptance within the game:

energy ball … if somebody is just like … throws it over their shoulder, we can all laugh at that and go, oh, that was their choice. Or if … somebody blows their nose on the imaginary thing and passes it on, the next person is like, oh, I’m not picking that up like that… if we are wishing them to engage in a performative way to honour the performance that they offer us in a very much of a sort of Theatresports-kind-of-rules way of accepting the offers that they put forth.

The team adapted this game to pass the ball across Zoom windows, finding playful ways to use the camera when simulating passing an object; it was even used in the project's hybrid capstone Encore Showcase (56). Louise (AF) described another warmup game that adapted well to Zoom, “One of the games we would play is ‘What body part?’ and this translated online… what part of your body is/wants to make a sound. And it was great.” Physical warmups helped the group become energized and be present.

Starting gently was another approach to help all team members enter into play in the Zoom environment. Robby (AF) described how this removed pressure and helped build trust:

Just kind of starting with some, you know, basic things like a little name game. And it was good to do gentle things like that, and gentle things that just let people contribute in a very, achievable manner and share something about themselves and that then kind of builds the trust. And once you have trust, then people are a little more available to be vulnerable, right?

It was important, “just to validate … the play impulse” (Esme, AF). Another technique used to achieve this, as well as help balance power relations (both in person and virtually), was for all team members to participate in CCE together. Esme (AF) described this approach,

we're all just rolling up our sleeves and getting in there, side by side, which is valuable. Which is something that we have been able to do on Zoom. Like for instance when we do a creative activity … everybody [each team member] gets to go.

Working together in this way helped minimize power hierarchies and helped to engender empathy and understanding, “There's no escaping play, … you understand the feelings that they [peer collaborators and care partners] might be having, because you also have to do it” (Sonia, R).

Another technique the team used, now that they were virtually sharing more intimate, personal space, was to draw inspiration for play from peer collaborator's home spaces and circumstances. Ki (AF) described how she suggested an activity based on a peer collaborator's and care partner's routines,

“One of our playful investigations was … they told me … they like to go for a walk outside their condo along the waterfront, …They took photos. And so it was like a photo adventure scavenger hunt. They took photos all along their walk. And then they brought it back to the Zoom call.”

Adam (AF) agreed that seeing intimate spaces contributed to creativity, “I think the bonus of Zoom space was …seeing homes.” Elizabeth (AF) added that seeing “garden spaces” became important to the project. In fact, the collaboratively composed “Protest Song” presented at the project's final Encore Panels (57) event used garden imagery (suggested by peer collaborators and care partners) in the lyrics of the song, and photos they took of their gardens in the accompanying video.

Not only did peer collaborators' spaces offer a window into their lives and contribute to playfulness, AFs' spaces were also visible and became part of the fabric of the interactions. Louise (AF) described how attending to her own surrounding space set the stage for playfulness and creativity,

I need to be in my studio. And I do Zoom because I'm surrounded by things that stimulate me, constantly. So, I've got beads in front of me, I've got things that I could draw on. Things I can play with, because that helps me bring it back to the moment.

Animals became a natural part of the Zoom environment and often offered inspiration and sometimes humour. Louise (AF) described the central presence of her dog, “She was essential. Like, when we did it, she was always [there]. She just became part of the virtual reality… and that became a real conversation starter. And then they started looking forward to it.”

While home spaces offered many advantages, peer collaborators and care partners lacked the wide range of art supplies that were available when the project met in person. Many team members missed working more extensively with tangible materials, as Louise (AF) expressed, “there's so much amazingness that can come from that [being online]. But for me, I'm always really grateful for something that I can do with my hands and without the glare of the screen.” To compensate for this RTC turned to mailing packages to peer collaborators, and when public health orders allowed, AFs sometimes drove packages to peer collaborators' houses. Louise (AF) describes this playful experiment,

the participants and our team are sending sort of creative care packages to each other, with art supplies in them… and, what's amazing is like, you can see what's been made so far, and then you're also left with, their kind of scraps… and you can add with your own materials that you have, and then send it on to somebody else.

Louise (AF) further explained that “it's not what you put in the package, it's the intention” and that the packages included “just like little things that would evoke, I don't know, play… like pieces of paper, different colours.” Sometimes team members opened the packages together online during creative engagements. Louise (AF) described the response of one recipient, “she just lighted up.” Being thoughtful about the content was important. The team learned that if the package contents were less intentional, “[peer collaborators and care partners] didn't have that same connection of what was going on in the bag. And so it didn't work for them” (Louise, AF).

#### 3.2.3. Maintaining social rituals

Finally, an important aspect of re-imagining CCE in virtual space was the team's concerted attempt to “maintain social rituals.” Prior to the pandemic, RTC recognized the importance of social rituals in providing social opportunities, creating a sense of connection, and helping with predictability. The project's most important social ritual was “tea and cookies.” Elizabeth (AF) said, “We always felt crunched for time. And regardless, we took tea and cookie breaks with our participants … just having that social time of being together … was always something that we prioritized.” James (AF) mentioned the particular social value that tea and cookies had for peer collaborators,

For so many people with a lived experience of dementia, … their social networks get smaller… it was just this social banter and teasing and that social connection, and just knowing that you're surrounded by friends, right? Like, I mean, it was a really, really, really great friendship circle.

It was not surprising, then, when peer collaborators raised the need to maintain this social ritual in a virtual context. Ki (AF) noted how peer collaborator Betty exclaimed during a creative engagement session “we need food and drinks or cookies.”

While the team found virtual CCE more effective and rewarding when done in smaller groups, RTC occasionally made a point of bringing the entire team together for tea or cookies or other celebratory events in order to maintain the social ritual of gathering as a community. During such sessions team members noticed how much peer collaborators enjoyed seeing each other for social time. Esme (AF) highlighted the sense of enthusiasm such virtual social rituals inspired, “our participants seemed to really enjoy group zooming … the twenty minutes of waving at each other and making jokes was so awesome.” Team members also often dressed up and brought a mug to match the theme of the occasion (for example, Halloween or Christmas) which inspired playfulness and connection.

One event the team organized was a virtual wrap party that took place after the online two-day livestream event Backstage Pass (54, 55). James (AF) spoke to the meaningfulness of this social ritual for peer collaborators and care partners, “In the wrap-up party, … you could really feel that sense of joy and celebration and accomplishment. Fatigue, for sure, but people are just laughing, and they're raising a drink… like, what's next?” Part of the reason these events worked with a larger group (when CCE did not) was that they did not take place too often, they were short in duration (less than an hour), and participation was very open-ended. People could join in conversation or just watch and they could leave whenever they wanted.

### 3.3. Sustaining reciprocal caring, learning, and support

A central value for RTC was that caring, learning, and support were mutual and reciprocal, and this became important to translate to virtual engagement. Reciprocity was central to the team's approach to relationships, as Elsie (R) described,

I think reciprocity … I don't know, like which one came first, reciprocity or like a sense of community, and a sense of belonging, but I think … attending to this principle helps us to establish a shared sense of community and sense of belonging.

Esme (AF) described how amongst AFs and researchers this meant sharing the lead during CCE, “care happens in the form of if you are in the lead or presenting/facilitating a certain workshop … your fellow artists, collaborators, researchers, whoever's in the room helping by supporting what you're doing.” She also noted how it was important as a leader to receive help by “being open to each other's assistance and observations.” This openness also extended to making space for peer collaborator's ideas and leadership, “It was completely essential to prioritise and to ask what the participants wanted to do … they have the creative power and initiative themselves to build what it is that they want to in the project” (Elizabeth, AF). It also meant recognizing peer collaborators' capacity to provide caring, support, and insights. James (AF) described how this is seldom recognized for people living with dementia,

SOOO MUCH, if they've gone through the traditional way people react to them … they become a burden, right? … So how do you build enough of a relationship and caring and assisting, that it's not a burden–and it's not a burden when it's reciprocal. Because you've done something for them. They'll do something for you and that balance, I think that's important.

Being reflexive about power relations and interactions was essential. AFs, such as Louise, described how this orientation toward reciprocity and mutual support and learning was part of re-imagining more typical ways of engaging with individuals living with dementia**,** “[it involved] stepping outside the clinical model [or traditional medical model], really. Set it aside.” DD (AF) further elaborated, “it's really in some ways dropping the role of client, it's interpersonal relationships, and a friendship. I never lost sight of my role, but it didn't define how I related with them [peer collaborators and care partners].” This meant that in Esme's role as an AF, she would “sometimes be a contributor and sometimes be a witness.” She further explained, “these are my elders in a community and I want to learn from them as much as I can.”

Part of what allowed this openness and reflexivity to continue to flourish in the shift to online engagement, was everyone's commitment to the project. Esme (AF) described the team's response to the virtual transition, “everyone involved in the project from like, team members, to peer collaborators, to care partners was very deeply committed.” For Elizabeth (AF) peer collaborators' commitment was evidence of how much they cared, “I just was so impressed with the bravery and the adaptability of our participants who felt like no, we still want to see you, and hear you, even if it's online, even if it's on Zoom.” She further elaborated on how support, understanding and adaptability went both ways in the shift to the Zoom world:

The care through technology … it was mutual and reciprocal and we didn't make them feel like a burden, … we were being introduced to zoom around the same time, you know. So we could empathize with the struggles. We were muted sometimes when we didn't mean to be. It bonded us. In a way because we were learning together.

The strength of the team's relationships established prior to the pandemic undergirded the project's ability to maintain reciprocal caring, learning, and support online, “thankfully we had had two and a half years of team building, so those relationships had been strong and formed. I'm not sure how easily that could have been done [working virtually] if we were starting from scratch” (Adam, AF). Many team members described how RTC's continued orientation toward reciprocal caring, learning, and support was deeply valuable to them. Esme (AF) detailed how, for her, offerings of reciprocal care reminded her of her own vulnerability and humanity,

I'm the facilitator, and I'm supposed to be caring for you … sometimes when you have the tension of needing to be there in a professional aspect, to be called into your human self [by a peer collaborator offering care and support] is really valuable, REALLY valuable.

The project's reciprocal orientation was highly valued by care partners too, as Tegan (CP) expressed,

It's given my mom peers. People who she feels safe around and who she IS safe around because so many people don't know how to interact in an empowering way with people who have dementia … she's safe and in an environment within a community.

Finally, DD (AF) commented on the uniqueness of this approach, especially in a virtual environment, “it's a structure that is specifically focused on care and support and listening … it's a structure that is unfortunately rare.”

### 3.4. Disrupting stigma and welcoming a wider audience

A strong focus of RTC was to disrupt the stigma of dementia. Elsie (R) explained,

[An] intention in RTC … was to intentionally disrupt the stigma of dementia, disrupting the stereotypes of people living with dementia as those lacking capacity and lacking agency… in RTC, particularly people with dementia, were seen as creators, as mentors, as teachers, as performers.

Part of disrupting stigma involved creating safe spaces to talk openly about it. Ki (AF) articulated the significance of this, “[if] you have the lived experience of dementia … you can feel unsafe … if you don't feel like you fit in, or that you belong, or you've been made to feel othered or left out.” She noted the effects this can have on people living with dementia, “either people treat them differently, or life is boring and repetitive, or there's actual harms that are happening to them.” The stigma of dementia was explored in depth from peer collaborators' perspectives using interviews, discussions groups, and creative engagements in an earlier phase of RTC (see Reid, Landy and Henderson) (63).

The shift to online engagement brought the opportunity to further challenge the stigma of dementia, and also the stigma that older adults would have insurmountable difficulties with technology. Elizabeth (AF) noted “maybe one or two have not transitioned to working on Zoom. But for a large percentage of our participants to convert to using Zoom at their age range, it blows me away.” The virtual transition provided peer collaborators and care partners with opportunities to learn new skills which were rarely offered to older adults prior to the pandemic. As James (AF) described, “it made people more comfortable with the technologies, … they wouldn't have learned those skills, … [RTC] gave that commitment, gave that focus … it set a framework.”

One novel aspect of RTC was that, from the beginning, it punctuated its ongoing CCE activities by doing live performances for audiences, in which peer collaborators participated in central roles. Prior to the pandemic these in-person events were for small invited audiences. A significant advantage of the project shifting online was that it could now expand its audience by using livestreaming and publicly posting recordings of events online; “we were able to share that with a bigger audience really, or a more worldwide audience, in our performance of Backstage Pass” (Elizabeth, AF). This type of public performance required bravery,

for our project, and our participants … to shift to a completely changed goal was incredible and surprising, and not surprising. I feel like our participants were so brave in the way that they shared their heart … [to] spend time on a live online event, … like how challenging that must have been for them (Elizabeth, AF).

The online performances went a long way in disrupting stigma. One audience member from Backstage Pass (55) wrote in the performance feedback,

One of the most profound impacts this project has had on me is it has expanded my imagination about what the “lived” experience of dementia could be—and most importantly, has chipped away the corners of my fears of getting dementia myself.

James (AF) described how the reach of the online performances influenced the international dementia community.

The team making that switch to Backstage Pass and going online … It inspired a whole other group of individuals like Gary Glazier and Susan McFadden in Milwaukee and the crew in the Timeslips organization, you know, for them to do their Memory Camp online because they saw this, it reached a much broader audience. That Dementia Lab, also, at Emily Carr tried to do it, so it opened up all these avenues.

For Joan (CP), challenging stigma publicly had great import, “I am just very, very grateful and I appreciate how Raising the Curtain is bringing this whole thing out into the larger community, larger world and the stigma is disappearing.”

### 3.5. Supporting well-being through empowerment, community, and creativity

RTC peer collaborators and care partners talked about the value the project had for them in terms of their well-being. When asked during a Backstage Pass livestream performance (54). “Why are we doing this? [RTC engagement],” Marguerite (PC) responded,

Well, there's a lot of people who have dementia, and often they're lost or abandoned, or whatever, as so having a program called Raising the Curtain with other people with dementia and people like you to facilitate it, we come to terms with our dementia in maybe more open ways, and we're with a group of people who also have dementia so you’re not alone, and you don't have those stigmas and the things that society dumps on you. And so that's very useful. And you feel pretty good about what you do!

Care partners also expressed how RTC helped empower families by redressing the stigma of dementia. Tegan (CP) noted that because of RTC,

Dementia was being talked about openly in our home and we were openly telling people that dementia is part of our lives. Whereas before there was a fear of telling people… having this community and doing the artistic engagement, I think, was very empowering

Leigh (CP) also expressed how RTC helped his spouse accept her diagnosis:

She's 88, and when she grew up dementia was “crazy” … When we started this program, she was saying, oh, she didn't like the word dementia at all… during the course of this program, she's accepted it. And that, to me, is a major change.

Having a safe and accepting community helped people feel less isolated, as Cheri (CP) expressed, “to be with other people that have the lived experience of dementia and that, it's so critical to connect with other people. So that we're not so alone in the diagnosis.” The sense of community was of great benefit to care partners as well as peer collaborators, “You don't just need community for your partner or a companionship for your partner, but there is a need for a community for both of you” (Joan, CP). Leigh (CP) conveyed the value that the accepting nature of the RTC community had for him,

I love the program for the humour and the acceptance and everything else, and seeing that part. That the person that's still there, but we also have to recognize, sometimes we have to cry together because it's very sad. Sometimes [it's a] very sad journey.

Leigh (CP) also noted that RTC helped expand his community and base of support, “I find that my community has expanded in very unexpected ways and it's been a wonderful journey.”

Care partners described how they felt that the creative engagement aspect of RTC was unique,

It is really the only thing we've heard about in Canada… I've not heard quite something like [this] … there's actually performing things for people to join and be together with and it's very, very crucial for people with dementia to be in. (Joan, CP)

Similarly, Sandra (CP) found RTC's approach to be novel and especially valuable,

I think that the creative engagement aspect of this is something that is very different to [other things] I take my mum to … [it] adds a whole other dimension to the stimulation.

Care partners highlighted the value of the caring nature of RTC's approach, “I'm totally blown away by [RTC] … how respectful it was” (Michael, CP). Tegan (CP) described how RTC created opportunities for advocacy and supported identity continuity for her mother, thus enhancing her well-being:

My mom, she's somebody who's always … been an advocate for all kinds of things in her life and that is a huge part of her identity. And so, for her to feel as though she could advocate for people with dementia was huge, for her to still maintain a sense of her strong identity and validation as an advocate.

RTC's CCE workshops promoted lasting positive emotions for all peer collaborators; “That feeling … that [it] creates within her just lasts her through the whole of the rest of the day and into the evening. And it's incredible to see” (Sandra, CP). For care partners, positive feelings lasted long after too, “everybody who was involved, caregivers as well, whenever it occurred, whenever there was activity, it was a really wonderful thing because you carried that with you for quite a while, often days” (Michael, CP).

While the transition to virtual engagement was felt to lack some of the power of in-person engagement— “Zoom is great and I’m glad you have it but it just doesn’t energize the same way” (Leigh, CP)— care partners and peer collaborators alike felt that it was effective and enhanced their feelings of connection and well-being. Sandra (CP) described the transition for her and her mom,

I would say it did actually work remarkably well. In the initial attempt when we had a screen that had, I don't know, 15–20 people on it didn't work very well because it was a little bit too confusing. But once we went into smaller groups with two facilitators and two participants with their caregivers, that worked really well and my mum was still in, me too… the degree of stimulation obviously was not the same. But it worked and it kept the connection ongoing … once the people appeared on the screen, she engaged with them completely, so it was great that that was able to carry on.

When peer collaborators were asked how they felt about participating by Zoom, Margaret (PC) responded, “it feels great, it feels easy and yes, it feels okay, it feels good.” Sadie (PC) expressed that for her it felt, “Just great! I enjoy people, meeting people and talking to people and listening to their lives. I just enjoy talking probably.” Leo (PC) felt connecting online was valuable, “to me even going through a system like we’re doing here today is a real goldmine in my thoughts.” Traudi (PC) described the impact of collaborating online with her daughter and husband on a dance/song/poetry piece, “real life or real love, real everything, everything REAL is what I shared in.”

Overall peer collaborators and their care partners expressed appreciation for the value and impact of RTC. When asked if the project played a role in improving quality of life for her mother and family, Tegan (PC), responded, “100%. Absolutely, absolutely.” Marguerite (PC) offered her encouragement to the RTC team to continue its virtual engagement, “Good for you. That's what you do when there's a pandemic and there's technology to help … it's a good approach.” Sandra (CP) summarised the valuable nature of the RTC project and online engagement,

I fully believe that without this kind of engagement, my mum's dementia would be more advanced. She steps up a level when with other people and is involved in an activity that is centred around her abilities.

Peer collaborators and their care partners spoke unanimously about the positive impact of their online involvement in RTC on their well-being through the team's ongoing focus on co-creativity, empowerment, and community building.

## 4. Discussion

Like other studies on the use of digital technologies involving persons with dementia and their caregivers (47, 51, 50, 53), our findings suggest that, while virtual participation was not a perfect replacement for in-person collaborative activities, virtual CCE offered meaningful connection and stimulation to all team members during the COVID-19 pandemic. This was especially true for peer collaborators with lived experience of dementia and their care partners who were are at greater risk of more extreme isolation. Participants in this sub-study felt that virtual CCE sessions offered important social connections, and even more so, generated chances to collaboratively engage from a place of imagination, creativity, improvisation, and play. AFs, Rs, PCs, and CPs, all described how RTC drew on processes of relationality, reciprocity, shared-ownership and co-learning, that were aligned with CBPR principles (56–58, 60, 61). These processes are also consistent with the relational focus and non-restrictive, broadly inclusive understandings of creativity encompassed in both co-creativity (40) and creative care (34) approaches. Layering in CBPR principles brought an attention to method and rigor and heightened our focus on knowledge translation, especially for a broad public.

RTC's virtual context had both drawbacks and benefits in terms of relational citizenship. The relational model of citizenship, Kontos, Grigorovich, and Colobong argue, “broadens understanding of creativity by foregrounding how capacities and senses of the body are central to creativity in everyday life for persons living with dementia” (45). Relational citizenship “foregrounds the reciprocal nature of engagement and the centrality of capacities, senses, and experiences of bodies to the exercise of human agency and interconnectedness,” according to Kontos, Miller and Kontos (32). The body, they contend, has a “pre-reflective capacity to inform and express distinctiveness and relationality” (p. 194), that persists in the face of cognitive losses such as occur with dementia. In RTC, on the one hand, all team members described a loss of collective group experiences and shared group feeling (or vibe) when not in person—the body's pre-reflective capacity to sense and respond to other bodies was challenged, to an extent, when bodies did not share physical space. However, RTC developed playful techniques to continue to draw on embodied know-how in a virtual context, such as adapted improvised movement, mime, breathwork, singing, and tactile activities, often finding ways to use the Zoom lens to enhance creative experimentation. The project also incorporated peer collaborators' previously-learned embodied expressions of creativity, for example, knitting, singing, drawing, and improvised chanting. These were featured in online public performances, along with in-depth conversations about the lived experience of dementia, creativity, and activism, and publicly demonstrated the “intrinsic capacities in persons living with dementia for creative self-expression” (54). Seeing peer collaborators and care partners in their home environments also allowed AFs to use details of their personal, physical space to inspire embodied expressions of creativity and relationality. For example, in one activity everyone found and shared treasured objects from around their home. RTC also did its best to compensate for the loss of in-person group connection by offering occasional group Zoom sessions for the purpose of sustaining social rituals like tea and cookies, post-performance celebrations, and holiday gatherings. Social rituals incorporated embodied and relational actions and responses (such as: toasting, eating and drinking, turn taking in conversation, mutual encouragement, joking and laughing) and were important to fostering relational citizenship in the virtual context.

In addition to challenges, AFs also described relational and creative benefits of the smaller CCE groups. They allowed for more intimate, deeper connections with peer collaborators and the inclusion of activities that catered to their specific interests, talents, and abilities. In addition, RTC's virtual shift expanded relational connections by including care partners, giving them a better understanding of their loved one's creativity, offering them emotional support, and deepening their own creative expression. Noteably, this decreased care partners' opportunities for respite, but care partners in our study found the supportive group experiences more valuable than time away from their loved one. Since RTC's virtual engagements largely took place during lockdown, they also expanded relationships in unexpected ways, involving family members and pets as a regular part of the social milieux. Future projects, then, might consider using a combination of in-person and virtual CCE activities as an optimal way to foster relational citizenship.

Play was central to RTC. Similarly, Zeilig et al. assert that the co-creative process can facilitate well-being through play. In “Dancing with Dementia”, Kontos et al. also highlight the importance of playfulness, describing how participants living with dementia expressed themselves playfully and imaginatively through improvised movement connected to a narrative (54). Further, Swinnen and de Medeiros argue that for persons living with dementia, play is a way to explore potential for expression, meaning-making, and relationship-building and often leads to expressions of joy (68). In RTC, as opposed to these other projects, participants did not share physical space. This necessitated the development of techniques to inspire and support *virtual* play. Key to this was choosing playful approaches with *intentionality*. Sometimes warmup activities needed to start more gently in the smaller group context. Validating the play impulse for individuals became increasingly important. AFs used aspects of peer collaborators' home environments (as seen through the Zoom window) to inspire playful activities and responses (for example, two pairs took photos of their gardens and used them as inspiration to co-write a song). The project also used snail mail to send creative materials to peer collaborators, being careful to choose package contents with intention based on knowledge of the person. AFs, PCs, and CPs opened the packages together over Zoom and used the contents to engage in playful and creative expressions supportive of relational citizenship. The experience brought playful “moments of catharsis and release” that allowed for expressions of vulnerability and sharing similar to those described by Zeilig et al. in their With All study. Also similar to Zeilig et al., RTC, sought “an inclusive and equalizing approach” to supporting well-being and agency, and found ‘fostering feeling in virtual space’ to be and effective means of doing so. Fostering feeling involved acknowledging and validating all emotions, including frustrations around social isolation and technology use. CCE techniques were used to explore, validate, and, in some cases shift, negative feelings.

The importance of shared leadership and equally valuing each person's contributions was central to RTC, just like the co-creative process noted by Zeilig, West and van der byl Williams (40). This was reflected in RTC's theme ‘sustaining reciprocal caring, learning, and support’ in which leadership went beyond providing guidance, instruction, or input, to developing equitable processes that fostered care and support. It was important within RTC to create opportunities for peer collaborators to share in expressions of leadership (for example one PC taught her AF to knit), as they equalized power differentials and supported relational citizenship. Zeilig et al. further note that agency can include “apparent passivity, not actively leading”. In RTC, this type of leadership (for all team members) was conceptualized as participating in quiet ways that contributed to group collaboration, coming and going from larger groups according to one's will, and sometimes acting as a witness and holding space.

The rapid development of video conferencing and virtual streaming platforms during COVID-19 allowed RTC to offer virtual live performances to an international audience, further expanding opportunities for peer collaborators to demonstrate and grow their relational citizenship. Dupuis et al. have argued that the arts can be used effectively to challenge dementia stigma by offering alternative narratives that “challenge dominate assumptions, foster critical reflection, and envision new possibilities for mutual support, caring, and relating” (27). In terms of theatrical performance more specifically, Zeilig and Burke note that, while the use of theatre and theatrical techniques to reshape perceptions of dementia have been explored by a number of scholars, they are “only recently being explored with people living with dementia as co-producers” (69). RTC, then, not only adds to the limited research on involving people with dementia in live performance, it also offers novel insights into how to do this virtually. Through involving peer collaborators and their care partners in live virtual performances [that involved high levels of creative expression, care, and education as Basting advocates (35)], RTC disrupted both dementia stigma and stigma around older adults and technology use, and inspired other creative dementia programs internationally to pursue virtual performance.

Navigating virtual engagement required sustained attentiveness to reflexivity (6, 56), especially given the power differential resulting from the fact that RTC researchers and AFs, on the whole, had more facility with, and access to, technology than its peer collaborators and care partners. Hebblethwaite et al. highlight the importance of attending to digital inequities such as internet access and ownership of digital devices, and the varied digital literacy of older adults (70). They note that desire to maintain social connection shifts when infrastructure is poor or initial experiences with digital technologies are unpleasant (p. 174). In RTC it was important, as a team, to thoughtfully and reflexively discover supportive approaches to technology and virtual engagement, such as those described in this article's themes, to creative positive experiences. An iPad was provided to a peer collaborator who did not have their own functioning technology, a large monitor was provided to another who needed it for accessibility, and phone calls were used for those who found on-screen time challenging. Ongoing coaching and support around technology was offered to care partners, AFs and Rs supported each other in learning the technology, and the entire team was committed to developing virtual CCE techniques that continued to support values of access and inclusion. Despite this, not all peer collaborators acclimatized to virtual CCE and several chose not to participate in the CCE workshops once the project shifted to virtual delivery.

Despite the large amount of rich qualitative data that informs this article, a primary limitation is the inequitable representation of team members' voices and experiences. This article represents a secondary analysis of data that were mostly gathered for other purposes. While it provides a rich and interesting view into the opportunities and challenges of CCE with individuals living with dementia, research participants are drawn upon unequally in the analysis. With the stresses of the COVID-19 pandemic and our awareness of the serious mental health risks of isolation and loneliness for the peer collaborators and their care partners, as a team, we prioritized sustaining CCE and the public performances and found ways to document these processes. Peer collaborators and their care partners discussed their general experiences with RTC in the public performances, however it is possible that they may have wanted to present a positive view to the public and might have been more hesitant to talk about negative aspects. An important consideration for future studies and programming is the need to explore condition-specific adaptations that can be made in virtual arts-based program delivery, particularly in attempting to work with diverse forms of dementia such as people living with non-memory-led dementias (for example, accommodating visuospatial challenges for people wanting to access online arts-based interventions who live with Posterior Cortical Atrophy, or behavioural challenges within Frontotemporal Dementia).

## 5. Conclusion

In the early days of the COVID-19 pandemic, there was the widespread belief that it was difficult, or even impossible, to engage virtually with people living with dementia because a dementia diagnosis was thought to limit meaningful participation, and older adults in general were prejudicially believed to have extreme difficulty managing technology. RTC pivoted almost immediately to continue its creative engagements online with people living with dementia, and in so doing offered novel and specific techniques for implementing CCE in virtual contexts. Project findings offer new perspectives on how virtual CCE not only has the potential to decrease loneliness and isolation and associated mental health risks for older adults living with dementia and their care partners, but also can promote well-being through the intentional use of reciprocal, supportive, and caring engagement processes. Indeed, the positive impact of RTC resulted from team members' commitment to reimagining virtual CCE with flexibility and adaptability. Our reimagining and sustainment of virtual CCE challenges stigmatizing representations of dementia and offers insights into the potential of virtual CCE to promote inclusion and enhance relational citizenship for individuals living with dementia and their care partners.

## Data Availability

The datasets presented in this article are not readily available because ethical approval of this study did not include the release of interview and focus group data, and it would be difficult to de-identify this data enough to ensure confidentiality. Some data used in this study are from publicly available YouTube videos (to which participants consented and chose to use their real names). These videos are publicly available at the YouTube video links included in the reference list (64–67).
